# Encoding of Arousal and Physical Characteristics in Audible and Ultrasonic Vocalizations of Mongolian Gerbil Pups Testing Common Rules for Mammals

**DOI:** 10.3390/ani13162553

**Published:** 2023-08-08

**Authors:** Yara Silberstein, Felix Felmy, Marina Scheumann

**Affiliations:** Institute of Zoology, University of Veterinary Medicine Hannover, Foundation, 30559 Hannover, Germany

**Keywords:** mammal, age, vocal ontogeny, individual signatures, sex, arousal, ultrasonic vocalizations, audible vocalizations, body weight, development

## Abstract

**Simple Summary:**

Mammals provide information about their emotional state and physical characteristics through vocal signals. Due to a similar vocal production apparatus across mammals, common rules for encoding this information are proposed. With increasing age/body weight, it is suggested that mammals utter elongated calls of lower frequency. With increasing arousal, it is proposed that mammal vocalizations become higher in frequency and longer in duration. However, in recent years an increasing number of mammalian species have showed discrepancies with these common rules. In this study, we investigated whether developing Mongolian gerbil pups fulfill the proposed common rules by recording vocalizations in a low and high arousal condition in four age groups. We discovered they produce three call types covering the audible (<20 kHz) and ultrasonic (>20 kHz) frequency ranges. Interestingly, the call types differed in the encoding of arousal state as well as physical characteristics and only partly fulfilled the common rules suggested for mammals. Thus, our results show that divergent encoding patterns do not only differ between species but also between call types within a species, indicating that rules to transmit information can be shaped by social, developmental, and environmental factors or different production mechanisms for vocalizations in certain frequency ranges.

**Abstract:**

In mammals, common rules for the encoding of arousal and physical characteristics of the sender are suggested based on a similar vocal production apparatus. In this study, we want to investigate to what extent vocalizations of developing Mongolian gerbil pups fulfill these rules. We recorded vocalizations of 28 Mongolian gerbil pups in four developmental stages using a separation paradigm, suggested to induce different arousal levels. For low arousal, a pup was placed in an arena isolated from its siblings and parents; for high arousal, the pup was additionally stressed through the simulation of a predator. An unsupervised cluster analysis revealed three call types: ultrasonic (USV), audible vocalizations (ADV), and transitions between both (USV-ADV). The USV and USV-ADV rate showed an age-dependent decrease, contrasting an age-dependent increase for ADVs. Vocal correlates for the encoding of arousal were found for USVs and of physical characteristics for USVs and ADVs. However, the pattern of encoding these cues differed between call types and only partly confirmed the common rules suggested for mammals. Our results show that divergent encoding patterns do not only differ between species but also between call types within a species, indicating that coding rules can be shaped by socio-ecological factors or call type specific production mechanisms.

## 1. Introduction

Vocalizations can encode a variety of information about the emotional state and physical characteristics of the sender due to physiological and anatomical variations of the vocal production system (e.g., [1,2,3,4,5]). According to the source-filter theory, vocalizations are produced by an air stream originating in the lungs, travelling through the larynx and the vocal folds and passing the supra-laryngeal vocal tract (e.g., [6,7]). The pressure of the air stream, the length, thickness, and tension of the vocal folds, as well as the shape and modulation of the supra-laryngeal tract affect the structure of the vocal output. Physiological variations linked to the emotional state of the sender (e.g., [8,9]) are suggested to affect the respiratory air stream, the laryngeal muscle tension, and the modulation of the supra-laryngeal tract, thereby modulating the amplitude, tempo, fundamental, and formant frequency of the vocalizations (e.g., [1,10]). Anatomical variations linked to the physical characteristics of the sender e.g., the length and thickness of the vocal folds and the shape of the supra-laryngeal vocal tract, affect the fundamental frequency and the formant pattern of vocalizations (e.g., [5,11]). Thereby, they can encode the sender’s identity, sex, age, and body size and weight (e.g., [12,13,14]).

The mammalian vocal production system is suggested to be an evolutionary highly conserved system, proposing universal coding rules for the expression of the emotional state and physical characteristics of the sender (e.g., [4,5]). Indeed, with increasing arousal the majority of investigated species increased call duration, call rate, fundamental, and formant frequencies (see for review: [3,15]). Physical characteristics such as sender identity are suggested to be mainly encoded by the formant frequencies representing the individual anatomical structure of the vocal apparatus (e.g., [5,6]). Age and sexual dimorphism are encoded in relation to variations in body weight and body size [12]. Thus, according to the acoustic allometry hypothesis larger individuals produce a lower fundamental frequency and formant pattern than smaller individuals [13]. Although there is support for these common rules, there is an increasing amount of evidence showing divergent patterns. For arousal, the reviews of Briefer [3] and Zimmermann et al. [15] summarize studies showing the opposite pattern for call duration and fundamental frequency. For the physical characteristics, few species increase, rather than decrease, the fundamental frequency of their calls with increasing age and body weight (e.g., [16,17,18]). Thereby, species producing high-frequency tonal calls rely more on the contour of the fundamental frequency than on formant frequencies (e.g., [19,20]). Thus, the divergent encoding pattern was not only found between species but also between call types of the same species (e.g., [14,21]). This questions whether the proposed common rules really are common across mammalian species. To identify factors affecting the communality of the proposed rules, further comparative studies investigating different mammalian species using similar experimental designs and analysis methods are needed.

Mammalian infants represent an interesting development stage for species comparison, because independent from the species social system or life style many mammalian infants are dependent on their parents in regard to protection, feeding, and for altricial species thermoregulation [22]. Moreover, the effects of body size and age can be observed in individuals during developmental changes. When infants get older, their lung capacity increases, allowing longer call durations, the vocal folds get thicker, expecting a lower fundamental frequency, and the length of the vocal tract increases, expecting lower formant patterns (e.g., [13,23,24]). Using short-term separation paradigms, mammalian infants of several species showed different changes in the vocal behavior or acoustic parameters between situations assumed to induce low and high arousal. For example, kittens produced the same call type [25], infants of the grey mouse lemur produced distinct call types [26], whereas piglets produced two different call types in different arousal levels during handling of the animals [21]. Thereby, the changes in the acoustic parameters between the arousal levels were not consistent. With increasing arousal, kittens [25] increased, infant grey mouse lemurs decreased [26], while piglets showed no change of call duration [21]. According to the frequency parameters, kittens and grey mouse lemur infants decreased [25,26], whereas piglets increased, their fundamental frequency with increasing arousal [21]. Interestingly, for piglets changes in harmonicity were not consistent between call types, showing an increase for screams but a decrease for grunts with increasing arousal [21]. These divergent patterns demonstrate that although many mammalian infants have similar needs, divergent patterns in the acoustic expression of these needs can already be measured at infancy. In this study, we want to investigate to what extent the proposed common coding rules to encode arousal and physical characteristics can be found in developing pups of Mongolian gerbils using the same experimental design we used previously in kittens and mouse lemurs [25,26].

Mongolian gerbils (*Meriones unguiculatus*) are an important animal model for human hearing research (e.g., [27,28]). Thus, findings on the encoding of social information during infancy might be relevant in interpreting the neurobiological ontogeny of the auditory system. Further, Mongolian gerbils are highly social and live in family groups consisting of a dominant monogamous couple and their non-breeding offspring. Pups are born altricial, open their eyes around day 17, and are weaned after four weeks [29]. For adult Mongolian gerbils, ten syllable types have been reported, consisting of calls in the pure ultrasound range (USVs) and calls in the audible range (ADVs) [30,31]. Kobayasi and Riquimaroux [31] reported that USV syllables differed in frequency contour, e.g., upward modulated, arched, and can be combined with ADVs. ADVs can be pure tonal calls containing several harmonics and can be intermixed with noise bursts. Thus, for ADV non-linear phenomena, such as deterministic chaos and subharmonics related to disturbed vocal fold vibrations, can be observed. For pups, research focused solely on USVs and only a limited description of their acoustic structures is available (e.g., [32,33]). For USVs, a decrease of call rate [32] and mid-range frequency [33] with age was reported. Interestingly, three androgen-level dependent sex-specific call types were documented for pups older than 17 days [33,34,35]. In the closely related fat-tailed gerbil, USVs and ADVs were also reported for pups [17,36,37]. Thus, it can be expected that pups of the Mongolian gerbil will utter USVs and ADVs. Interestingly, for rodents two different vocal production mechanisms to produce USVs (aerodynamic mechanism) and ADVs (vocal fold vibration; e.g., [38,39]) are suggested, which might affect the encoding of social information [40].

The aim of this study was to investigate whether the occurrence and the acoustic structure of vocalizations encode the (1) arousal and (2) developmental state as well as (3) the individual cues (identity, sex) of Mongolian gerbil pups. Additionally, we investigated whether the changes of the acoustic parameters follow the common rules proposed for the encoding of arousal and physical characteristics in mammals. According to the common rules of encoding arousal, we hypothesize that pups will increase the call duration and fundamental frequency of their calls with increasing arousal. According to the acoustic allometry hypothesis, we expect that pups will increase call duration and decrease the fundamental and dominant frequency of their calls with increasing body weight and age. Further, we propose that sender identity is dominantly encoded by filter-related parameters due to individual variations of the supra-laryngeal vocal tract. Since immature pups show no sexual dimorphism in body weight, we expect no differences in the acoustic parameters between sexes.

## 2. Materials and Methods

### 2.1. Animals and Housing

We tested 28 Mongolian gerbil pups (14 males, 14 females) from seven litters and six different breeding pairs between the ages of 10 to 25 days. The pups were born in the breeding colony of the Institute of Zoology of the University of Veterinary Medicine Hannover, Foundation, Hannover, Germany. To refresh the genetics of the colony, animals from Charles River (Sulzbach, Germany) are regularly added. All animals lived in their litter with their parents in a macrolan cage (610 × 435 × 215 mm), being weaned at one month of age. The cage was lined with approximately seven cm of wood shavings and equipped with a wooden nesting box. Pellets (SSNIFF Spezialdiäten GmbH, Soest, Germany, Complete diet for gerbils—10 mm) and water were offered ad libitum. Every other day, a few pieces of vegetable and a tissue for nest building were offered. The room was occupied with breeding pairs and their pups and had a temperature of 22 ± 2 °C, relative humidity of 47 ± 7%, and a light/dark cycle of 12:12 h (lights on at 07:00).

### 2.2. Experimental Set Up

The set up was assembled in a semi-soundproofed chamber with an approximate 20 cm diameter round wire mesh cage placed on a towel to prevent the pups from leaving the experimental set up. Vocalizations were recorded using a microphone (MKH 8020; frequency range 10 Hz–70 kHz, Sennheiser electronic GmbH and Co. KG, Wedemark, Germany) linked to a Zoom F4 or F6 Multitrack Field Recorder (K.K. Zoom corporation, Chiyoda, Tokyo, Japan). The audio files were recorded using a sampling frequency of 192 kHz and saved as .wav files. Moreover, the presented behavior was recorded by a digital camera E1 (Reolink, Compton, CA, USA) connected to a Synology surveillance system (Synology Inc., New Taipei City, Taiwan).

### 2.3. Experimental Procedure

A pup was separated from its parents and siblings and transferred in a transport box from the housing cage to the experimental set up in the semi-soundproofed chamber. It was exposed to two different conditions, which were supposed to induce different arousal states (e.g., [25,26]). In the Low arousal condition, the pup was placed alone and undisturbed on a paper towel surrounded by a wire-mesh cage. Since the pup did not move or show exploration behavior, we assumed that this isolation context induced a lower arousal state of negative valence than the High arousal condition. In the High arousal condition, the pup was handled by the experimenter, turned on its back, lifted off the table and softly poked. Since the pup struggled, tried to turn back, and tried to avoid the experimenter, we assumed that the handling induced a higher arousal state of negative valence than the Low arousal condition. Each condition was carried out for three minutes. After all conditions were tested the pup was weighed and the state of eye opening was noted (open: both eyes were fully open, closed: both eyes were fully closed, half opened: both eyes were not fully open). Finally, the pups were reunited with their parents and siblings in their housing cages. To investigate whether vocal behavior in the two arousal conditions change across postnatal development, pups were tested in four age groups: 10–13 days (mean = 11.3 d.), 14–17 days (mean = 15.3 d.), 19–21 days (mean = 19.4 d.), and 23–25 days (mean = 24.7 d.) post natum, reflecting different developmental stages. In each age group, the order of tested pups as well as the order of conditions were randomized.

### 2.4. Acoustic Analysis

Using Audacity (Free Software Foundation, Inc., Version 3.3.2, Boston, MA, USA, www.audacityteam.org) we visually inspected the spectrograms of audio recordings for pup vocalizations. We detected three potential call types covering three different frequency ranges: calls in the ultrasonic frequency range (USV), calls in the audible frequency range (ADV), and calls in both frequency ranges (USV-ADV). To quantify the number of calls, we counted the calls manually by visually screening the spectrograms of the audio files of each individual, condition, and age group.

**Figure 1:**
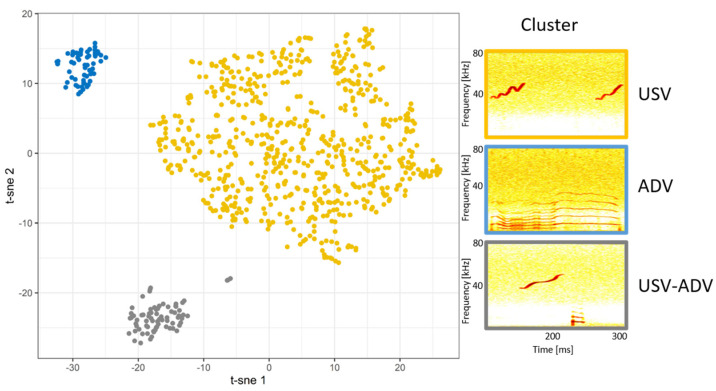
t-SNE plot visualizing results of unsupervised cluster analysis. Colors represent separate clusters matching the preliminary visual classification of the call types; USV—ultrasonic vocalization, ADV—audible vocalization, USV-ADV—transition from USV to ADV part.

For the acoustic characterization, we selected the first 10 good quality calls (high signal-to-noise ratio, not clipped), if applicable, for each individual in each age group, each condition, and each potential call type from the detected calls (N = 26,798 calls) in order to balance the data set. In total we measured 817 calls, which consisted of 680 USVs (n = 26 pups), 61 ADVs (n = 13 pups), and 76 USV-ADVs (n = 13 pups) (Figure 1). We performed a multi-parametric sound analysis using PRAAT (Version 6.1.12, Phonetic Sciences, University of Amsterdam, The Netherlands [41]) combined with GSU Praat Tools 1.9 scripts [42]. First, we pre-processed the audio files by band-pass-filtering them according to their frequency ranges (USV: 20,000–100,000 Hz; ADV: 100–50,000 Hz; USV-ADV: 500–70,000 Hz) to improve signal-to-noise ratio. For each call, 16 acoustic parameters (three time-related, six source-related, four filter-related, and three tonality-related parameters) were measured (Table 1): call duration (Dur), time of minimum frequency (TimeminF0), time of maximum frequency (TimemaxF0), minimum fundamental frequency (MinF0), maximum fundamental frequency (MaxF0), bandwidth (BandF0), mean fundamental frequency (MeanF0), standard deviation of fundamental frequency (SDF0), meanslope (SlopeF0), center of gravity (CoG), standard deviation of the center of gravity (SD), skewness (Ske), kurtosis (Kur), percentage of voiced frames (Voiced), harmonics-to-noise-ratio (Hnr), and wiener entropy (Entropy [43]). For the pitch tracking, we extracted the F0 contour using the To Pitch (cc) command in Praat, compared the extracted pitch contour with the sonagram, and corrected the tracking manually if necessary. Due to the different frequency ranges of the calls, we had to adapt the tracking settings (USVs: pitch floor: 20,000 Hz; pitch ceiling: 70,000 Hz; ADVs: pitch floor: 2000 Hz; pitch ceiling: 25,000 Hz; USV-ADVs: pitch floor: 500 Hz; pitch ceiling: 70,000 Hz; time frame = 0.003 s for all). For USV-ADVs, minimum and maximum fundamental frequency were not always tracked very well due to the large frequency range. Therefore, we performed an additional pitch tracking for the USV and ADV parts separately and combined the measurements. Thus, measurements of the maximum fundamental frequency refer to the MaxF0 and TimemaxF0 of the USV part, whereas measurements of the minimum fundamental frequency refer to the MinF0 of the ADV part. TimeminF0 was calculated for USV-ADVs from the onset of the call until the Time of minimum fundamental frequency in the ADV part. Further, for USV-ADVs percentage of voiced frames (Voiced) was calculated by summing the voiced and unvoiced frames of the USV and the ADV parts to exclude the intersyllable-interval between both parts.

### 2.5. Statistical Analysis

#### 2.5.1. Validation of Different Call Types

To investigate whether the visually classified potential call types could be mathematically determined, we performed an unsupervised cluster analysis. First, we standardized the acoustic parameters using z-transformation. Second, we tested them for independence using a pairwise Pearson correlation test. Hence, if two parameters resulted in a correlation coefficient >0.7, we maintained only one of them for the cluster analysis. Third, we performed a K-means clustering (e.g., [44,45]) using the Silhouette method (repetition = 500, random starts = 100, maximum possible clusters = 100). An elbow figure was produced which automatically decided the optimal number of clusters. A two-dimensional visualization of the resulted clusters was plotted by a t-SNE analysis ([46]; Figure 1).

#### 2.5.2. Effect of Arousal, Sex, and Age on Vocal Behavior

To prove whether our assumption that body weight increased with age but did not differ between immature sexes, we performed a Linear Mixed-Effects Model (LME) using Body weight as test variable and Sex, Age group, and the interaction of both as predictor variable while controlling for Identity.

To investigate the effect of the arousal condition, the sex, and the age of the sender of the vocal behavior, binomial Generalized Linear Mixed-Effects Models (GLMM) for call occurrence and LME models for call rate were calculated for each call type. For the full model, we used call occurrence (yes, no) or call rate as test variable, Arousal condition (low, high), Sex (male, female), Age group (10–13 d., 14–17 d., 19–21 d., 23–25 d.), and Order of condition (first, second), as well as the interaction between Arousal, Sex, and Age group as predictor variables, and Sender identity nested in Litter as random factor (GLMM: Call occurrence ~ Arousal * Sex * Age group + Order + (1|Litter/Identity); LME: Call rate ~ Arousal * Sex *Age group + Order + random = ~1|Litter/Identity). Based on the full model, the best fitting model was established using stepwise backwards elimination [47]. At each step we reduced the highest-level interaction term with the highest non-significant *p*-value and compared the previous one to the reduced model using Wald test statistics (‘anova’ command). Finally, the elimination procedure was stopped when (1) the Wald test indicated a significant difference between the reduced and the previous model selecting the previous model, or (2) only main terms, or (3) main terms and significant interactions remained in the final model. In the result section we only reported on the final models. If the final model contained significant interaction terms, we conducted a break-down analysis, splitting the dataset to analyze the factors separately.

In Age group 14–17 d., the status of eye opening varied between individuals, allowing us to test for an effect of eye-opening independent from age. Thus, we performed additional GLMM and LME models for Age group 14–17 d. using call occurrence/call rate as test factor, Arousal and Eye opening (opened, closed, half opened) as predictors, and Identity nested in Litter as random factor.

#### 2.5.3. Vocal Correlates of Ultrasonic and Audible Vocalizations

We investigated whether arousal, developmental factors (body weight, age group), and individual cues (sender identity, sex) are encoded in pup vocalizations. The analysis of the effect of arousal on call structure was limited to USVs, because only USVs occurred in both conditions in a sufficient sample size. Therefore, we calculated LME models to test whether the acoustic parameters differ between the two arousal conditions by controlling for Identity nested in Litter as random factor (LME: Acoustic parameter ~ Arousal + random = ~1|Litter/Identity). To control for multiple testing, a Fishers Omnibus test was performed [48]. To investigate whether the arousal condition can be statistically classified based on the acoustic parameters, we performed a permutated discriminant function analysis (pDFA; [49]) using Arousal as test factor and Identity as control factor. The pDFA balanced the contribution of control factors to the model by using the same number of calls for each individual in order to establish the model. Thus, to provide a sufficient training sample and to balance the number of calls per individual, individuals with less than six calls were excluded from the analysis as well as non-significant acoustic parameters. We standardized and selected non-correlating acoustic parameters as described above.

To investigate the effect of developmental factors and individual cues on the acoustic parameters of USVs and ADVs, we performed LMEs using the acoustic parameter as test factor, Body weight or Age group as predictor variable, and Identity nested in Litter as random factor for each acoustic parameter (Acoustic parameter ~ Body weight/Age group + random = 1|Litter/Identity) pooling the calls of both arousal conditions. If the predictor had only two levels or consists of metric data (body weight), we reported the estimates and standard error together with the results of the *t*-test. If the predictor had more than two levels, we reported the results of the ANOVA of the model.

To investigate individual cues, we tested whether the sender identity or sex affect the acoustic structure of pup USVs and ADVs. To test for individual distinctiveness, we performed LME models using the acoustic parameters as test variable and the Identity as predictor variable while controlling for Litter as random variable (Acoustic parameter ~ Identity + random = ~1|Litter) pooling calls of both arousal conditions. To test for sex, we performed LMEs using the acoustic parameter as test variable, Sex as predictor, and Identity nested in Litter as random variable (Acoustic parameter ~ Sex + random = ~1|Litter/Identity). Based on the significant acoustic parameters, a stepwise discriminant function analysis was performed to investigate whether individuals could be correctly classified. To balance the sample size, pups that produced at least five good quality calls were selected (n = 23). For each individual, a binomial test was performed comparing the correct classification against chance level. For sex we performed a pDFA using Sex as test factor while controlling for Identity using significant non-correlating acoustic parameters. To provide a sufficient training sample, individuals with less than six calls were excluded from the analysis.

The analyses were carried out in R (Version 4.1.0); accessed using RStudio (Version 1.4.1717); packages: t-SNE—“Rtsne” (Version 0.15); K-means model—“cluster” (Version 2.1.2); script for elbow figure from Romero-Mujalli, et al. [45]; graphical illustrations—“ggplot2” (Version 3.3.5); pDFA—“DFA.CANCOR” (Version 0.2.5), “stats” (Version 4.1.0), “psych” (Version 2.2.5); GLMER and LME—“lme4” (Version 1.1-27.1), “nlme” (Version 3.1-152), “car” (Version 3.0-11), “stats”(Version 4.1.0); while the discriminant function analysis as well as the binomial test were executed using IBM SPSS Statistics (Version 28.0.0.0).

## 3. Results

### 3.1. Distinction of Different Call Types

Based on the correlation analysis, the following ten non-correlating acoustic parameters were used in the cluster analysis (Figure S1): Dur, TimemaxF0, MinF0, MaxF0, BandF0, SlopeF0, SD, Kur, Voiced, and Hnr.

The silhouette model obtained the optimal number of three clusters (Figure S2), separating the calls into three different call types (Table 2). Cluster I matched the USV-ADVs, containing calls with a low and a high frequency part separated by a distance of <51 ms. Cluster II matched the USVs containing calls with a frequency above 20 kHz, with an upward sinusoidal modulated frequency. Cluster III matched the ADVs containing calls covering a frequency range of 2.3 to 14.8 kHz. Visualization of the calls using the t-SNE method confirmed the results of the cluster analysis (Figure 1) and the visual classification.

### 3.2. Effect of Arousal, Age and Sex on the Vocal Behavior

In general, all 28 pups produced vocalizations throughout the experiments. All pups produced USVs, 16 pups produced ADVs, and 13 pups produced USV-ADVs. As expected, Body weight increased with Age group (χ^2^ ≥ 1092.46, *p* < 0.001) but was not affected by Sex (χ^2^ = 0.78, *p* = 0.377).

**Figure 2:**
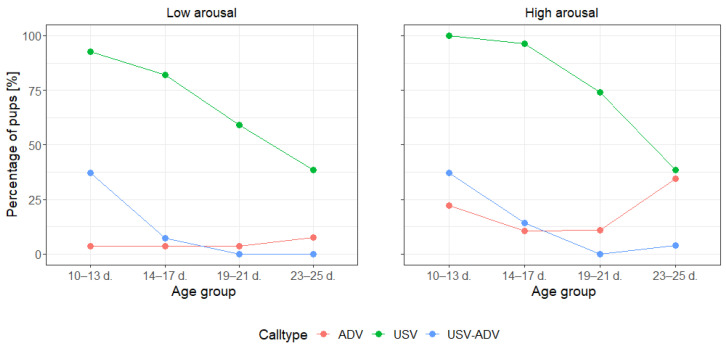
Percentage of pups producing ADVs, USVs and USV-ADVs in the Low and High arousal condition across Age groups; d. = postnatal days.

Analyzing the call occurrence, the best fitting model included only the main terms for all call types (Table 3, Figure 2). For the USVs, we found a significant decrease in the number of vocalizing infants with Age group (χ^2^ = 35.36, *p* < 0.001), but no effect of Arousal, Sex, and Order. For the ADVs, significantly more pups produced this call type in the High versus Low arousal condition (χ^2^ = 10.33, *p* = 0.001), but there was no effect of Age group, Sex, and Order. For the USV-ADVs, we found an effect of Arousal and Age group (χ^2^ ≥ 5.43, *p* ≤ 0.020) but no effect of Sex and Order. Thus, more pups produced USV-ADVs in the first two age groups, whereas no USV-ADVs were produced in the last two age groups. Additionally, significantly more pups produced USV-ADVs in the High compared to the Low arousal condition.

Analyzing the call rate, the final models contained the main terms but also an interaction for all call types (Table 3, Figure S3). For USVs and USV-ADVs, the model revealed a significant effect of Age group (χ^2^ ≥ 11.33, *p* ≤ 0.010), also showing a significant interaction between Age group and Sex (χ^2^ ≥ 7.92, *p* ≤ 0.048). However, the break-down analyses revealed that both sexes showed a significant decrease in the USV and USV-ADV rates across age groups (χ^2^ ≥ 10.28, ≤ 0.016). For ADVs, the final model showed a significant effect of Age group and Arousal (χ^2^ ≥ 6.24, *p* ≤ 0.013) as well as a significant interaction between Age group and Arousal (χ^2^ = 14.48, *p* = 0.002). The break-down analysis exposed that call rate increased with age in the High (χ^2^ = 16.67, *p* < 0.001) but not in the Low arousal condition (χ^2^ = 3.05, *p* = 0.384).

In Age group 14–17 d., the status of eye opening differed between the pups. GLMM models showed a significant effect of Eye opening and Arousal on call occurrence of USVs (*p* ≤ 0.012). However, neither Arousal nor Eye opening showed a significant effect on call occurrence for the two other call types nor for call rate.

### 3.3. Vocal Correlates of Ultrasonic and Audible Vocalizations

#### 3.3.1. Arousal

**Figure 3:**
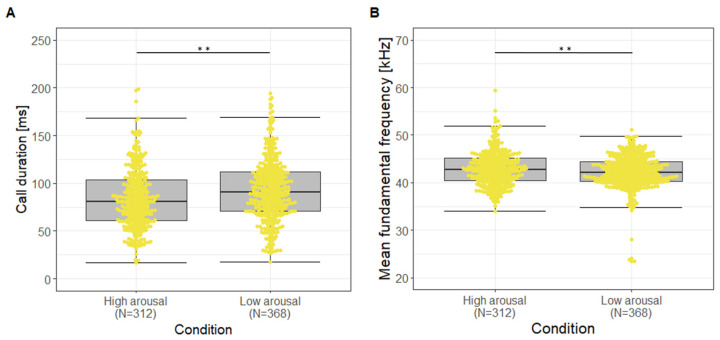
Boxplots for (**A**) Call duration and (**B**) Mean fundamental frequency of USVs during High and Low arousal condition. Boxplots represent lower and upper quartile, thick black line is the median, whiskers are the non-outlier range, yellow dots represent vocalizations plotted as a beeswarm, ** *p* < 0.01.

In contrast to USV-ADVs and ADVs, only USVs occurred in both arousal conditions in a sufficient number. For the USVs, LME analyses revealed that 11 out of 16 parameters differed significantly between the Low and High arousal condition (Fisher Omnibus test: χ^2^ = 165.09, df = 32, *p* < 0.001, Table S1). The time-related acoustic parameters showed a shorter duration and a shorter time until minimum fundamental frequency for the High versus the Low arousal condition (t ≥ 2.46, *p* ≤ 0.014, Figure 3A). The source-related spectral parameters MinF0, MaxF0, MeanF0, and SDF0 were lower in the Low versus High arousal condition (t ≤ −2.80, *p* ≤ 0.005; Figure 3B). For the filter-related spectral parameters, CoG was also lower in the Low versus High arousal condition (t = −4.97, *p* < 0.001), whereas SD and Ske showed the opposite pattern (t ≥ 2.57, *p* ≤ 0.011). The tonality of the calls was lower in the High versus the Low arousal condition (Hnr: t = −2.42, *p* = 0.016; Entropy: t = 2.87, *p* = 0.004). However, a pDFA based on the seven non-correlating acoustic parameters, Dur, MinF0, TimeminF0, MaxF0, Hnr, Ske, and SD, was not significant either for the original calculation (60% correct versus 57% chance level, *p* = 0.155) nor the cross validation (56% correct versus 52% chance, *p* = 0.144), demonstrating that the calls could not be correctly classified to the respective arousal condition.

#### 3.3.2. Developmental Factors

To investigate the impact of ontogeny, we used body weight and age group as a proxy for physical maturation. For USVs, only the first three age groups could be compared because pups almost stopped vocalizing in Age group 23–25 d. For ADVs, the first three age groups were combined (10–21 d.) to test them against Age group 23–25 d., because pups did not produce enough ADVs in these age groups.

**Figure 4:**
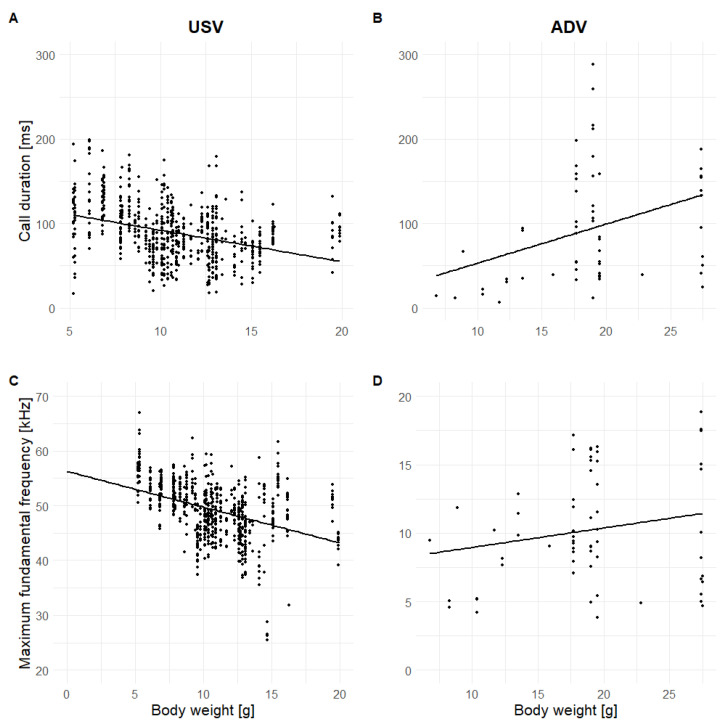
Scatterplots for (**A**) USV Call duration, (**B**) ADV Call duration, (**C**) USV Maximum fundamental frequency, and (**D**) ADV Maximum fundamental frequency across body weight. Dots = vocalizations, thick line = regression line.

**Figure 5:**
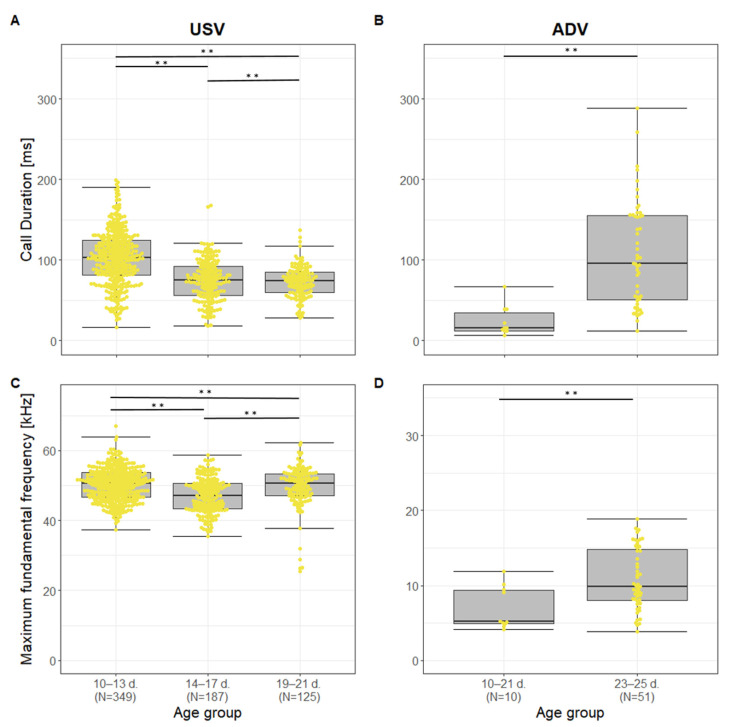
Boxplots for (**A**) USV Call duration, (**B**) ADV Call duration, (**C**) USV Maximum fundamental frequency and (**D**) ADV Maximum fundamental frequency across different Age groups. Boxplots represent lower and upper quartile, thick black line is the median, whiskers are the non-outlier range, yellow dots represent vocalizations plotted as a beeswarm, d. = postnatal days; ** *p* < 0.01.

For USVs, the same nine acoustic parameters were significantly affected by body weight and age as well as one additional parameter for body weight (SD) and three additional parameters for age (BandF0, Voiced, Hnr; Fisher Omnibus test: Body weight: χ^2^ = 174.13, df = 32, *p* < 0.001; Age group: χ^2^ = 386.25, df = 32, *p* < 0.001; Tables S2 and S3). The temporal-related parameters Dur and TimemaxF0 decreased with Body weight and across Age groups (Body weight: t ≤ −8.12, *p* ≤ 0.001; Age group: χ^2^ ≥ 130.04, *p* < 0.001; Figure 4 and Figure 5). For the source-related parameters, the frequency parameters decreased with Body weight (t ≤ −5.60, df = 653, *p* < 0.001), whereas the modulation of the fundamental frequency increased (t ≥ 2.31, df = 653, *p* ≤ 0.021; Figure 4). Interestingly, across Age groups a similar decrease in MinF0 and MeanF0 and a similar increase in SlopeF0 (χ^2^ ≥ 50.54, df = 2, *p* ≤ 0.001) were found, whereas MaxF0 and SDF0 first showed a decrease from Age group 10–13 d. to 14–17 d. followed by an increase from Age group 14–17 d. to 19–21 d. (χ^2^ ≥ 40.42, df = 2, *p* < 0.001; Figure 5), which corresponds to the same pattern as BandF0 (χ^2^ = 44.32, df = 2, *p* < 0.001). For the filter-related parameters, we found a decrease in the CoG and Ske with increasing Body weight and Age group (Body weight: t ≤ −2.11, df = 653, *p* ≤ 0.035; Age group: χ^2^ ≥ 7.59, df = 2, *p* ≤ 0.022). The SD was only affected by Body weight, but not by Age group (Body weight: t = 4.23, *p* < 0.001). Tonality-related parameters showed an increase of tonality across Age groups (χ^2^ ≥ 12.61, df = 2, *p* ≤ 0.002 for Voiced and Hnr), but no effect of Body weight.

For ADVs, only three acoustic parameters were significantly affected by Body weight and Age group, whereas four further parameters were significantly affected by Age group (Fisher Omnibus test: Body weight: χ^2^ = 52.74, df = 32, *p* = 0.012; Age group: χ^2^ = 78.73, df = 32, *p* < 0.001, Tables S2 and S3). The time-related parameters Dur and TimeminF0 increased with increasing Body weight and Age group (Body weight: t ≤ 2.26, df = 47, *p* ≤ 0.029; Age group: t ≥ 2.69, df = 47, *p* ≤ 0.010; Figure 4 and Figure 5), whereas the tonality-related parameter Voiced decreased (Body weight: t = −2.98, df = 47, *p* = 0.005; Age group: t = −2.30, df = 47, *p* = 0.026). For Age group, the source-related parameters MaxF0 (Figure 4), BandF0, and SDF0 increased (t ≥ 2.06, df = 47, *p* ≤ 0.045), and the filter-related parameter Ske decreased with increasing Age group (t = −2.48, df = 47, *p* = 0.017).

#### 3.3.3. Individual Cues

Analyzing individual distinctiveness for USVs and ADVs, all 16 acoustic parameters differed significantly between individuals (Fishers omnibus test: χ^2^ ≥ 357.20, df = 32, *p* < 0.001, Table S4). For USVs, the step-wise DFA selected nine parameters to calculate the model in the following order: CoG, SD, Entropy, Ske, Kur, SlopeF0, TimemaxF0, MeanF0, and Voiced. Calculating nine discriminant functions, 43.9% of the USVs were correctly classified (cross-validation: 37.4%; chance level: <9%). Binomial tests confirmed a correct classification for 22 out of 24 individuals. For ADVs, only four pups contributed with a sufficient number of calls for a DFA. Thus, we conducted no further analysis due to the low sample size.

**Figure 6:**
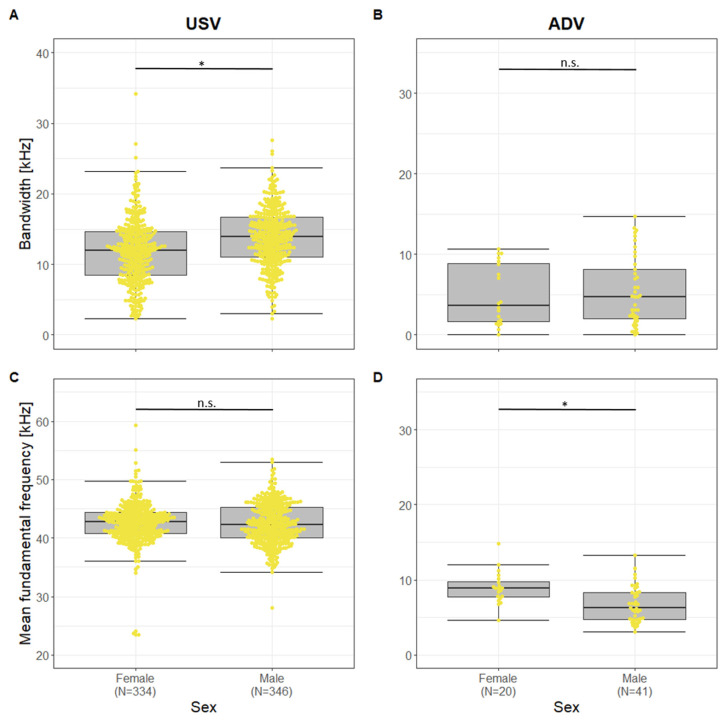
Boxplots for (**A**) USV Bandwidth and (**B**) ADV Bandwidth, (**C**) USV Mean fundamental frequency and (**D**) ADV Mean fundamental frequency for females and males. Boxplots represent lower and upper quartile, thick black line is the median, whiskers are non-outlier range, yellow dots represent vocalizations plotted as a beeswarm, * *p* < 0.05, n.s. = non-significant differences.

Analyzing sex-specific differences, different parameters were affected by sex for USVs and ADVs (Fisher Omnibus test: USV: χ^2^ = 48.06, df = 32, *p* = 0.034; ADV: χ^2^ = 60.00, df = 32, *p* = 0.002, Table S5). For USVs only BandF0 showed a significant difference between sexes. This parameter was higher in males than in females (t = 2.32, df = 18, *p* = 0.032; Figure 6). For ADVs, sex affected four parameters significantly (TimemaxF0, MinF0, MeanF0, and CoG; t ≤ −2.46, *p* ≤ 0.049). All four parameters were higher in females than in males (Figure 6). Since the significant parameters were highly correlated, resulting in only one non-correlating parameter, no pDFA was calculated for both call types.

## 4. Discussion

Our results showed that calls of Mongolian gerbil pups encode arousal and physical characteristics (for summary Table 4). Pups produced three call types (USVs, ADVs, USV-ADVs), covering a broad frequency range. The occurrence and call rate of these call types differed depending on the age of the subject and/or arousal condition. For USVs, which occurred in both arousal conditions, moderate variations in the acoustic parameters between arousal conditions were detected. The developmental state was encoded in the acoustic parameters of both USVs and ADVs but showed an opposite pattern for call duration and maximum fundamental frequency. The acoustic parameters of USVs and ADVs differed between individuals, and for USVs sender identity was correctly classified based on filter- and source related acoustic parameters. Although the pups were not fully matured, sex-specific differences in the acoustic structure between sexes were found for USVs and ADVs but were not consistent across call types. Thus, the vocal correlates of arousal and physical characteristics did not always follow the common rules suggested for mammals.

### 4.1. Function of Pup Vocalizations

The vocalizations of Mongolian gerbil pups uttered during isolation covered a wide frequency range producing USVs and ADVs, even at early developmental stages, similar to other rodents (e.g., fat-tailed gerbil: [17], hamsters: [50]; yellow-steppe lemmings: [18]; deer mice but not *Mus* sp.: [51]). Comparing pup vocalizations with the adult repertoire revealed that, according to their frequency contour, pup USVs correspond to the uSFM syllables of Kobayasi and Riquimaroux [31] and the Warble of Holman and Seale [33] but are shorter and have a higher minimum fundamental frequency. The occurrence and call rate of pup USVs decreased with increasing age which is in accordance with De Ghett [32], who also documented a decrease of call rate in pups from day 4 to day 20. Thus, with the independence of the pups from their caregivers, attention calls directed to them decreased. In other rodents, a decrease of isolation calls with age was documented (e.g., [36,52,53]). Thereby, in Mongolian gerbils the peak of USV production seems to be delayed in comparison to other rodents (vole, rat, mouse, Syrian hamster), which is suggested to be a result of the later eye opening [52]. This is supported by our finding that eye opening in the same age group has an effect on the occurrence of USVs. ADVs correspond to the audible syllables reported by Kobayasi and Riquimaroux [31] and the audible calls reported by Ter-Mikaelian, et al. [30] for adult Mongolian gerbils. Thereby, Kobayasi and Riquimaroux [31] distinguished four different audible syllable types (DFM1, DFM, QCF, NB). However, after performing a separate unsupervised cluster analysis only for ADVs, we found no further distinction of ADV clusters. This discrepancy can be explained by the restricted isolation context or by different analytic methods. Thus, also in the multidimensional scaling analysis of Kobayasi and Riquimaroux [31], the four clusters of the audible syllables showed a strong overlap. Pup ADV rate showed an increase with age in the High arousal condition. In the High arousal condition, the experimenter was bitten by two animals in the last two age groups. Thus, it could be assumed that ADVs might function as a warning to predators or induce aversive behavior to conspecifics [54] instead of eliciting parental care. Interestingly, transitions between USVs and ADVs occurred, which have only been described by Kobayasi and Riquimaroux [31] in adult Mongolian gerbils, showing that these transitions were not solely a result of the maturation of the vocal production system. To date, the function of these transitions is not clear. While adult ADVs are suggested to be associated with aggression, disturbance, and food dispute [30], pup USVs are suggested to elicit the attention of the caregiver (e.g., [55,56]).

### 4.2. Vocal Correlates of Arousal and Physical Characteristics

The hypothesis that, with increasing arousal, the call duration and fundamental frequency increase was not fulfilled in general. Despite this, for USVs, acoustic parameters differed between both arousal conditions, and pup USVs followed the common rules in relation to the fundamental frequency, but not to duration. However, a decrease of duration with increasing arousal within the same call type was also documented in other mammalian species (e.g., rodents: [57,58], primates: [59], scandentia: [60], carnivore: [61]). Comparing our results with other rodent studies using a similar design (Isolation refers to the Low arousal condition; Handling refers to the High arousal condition), we found that the investigated rodents produced USVs in both, the Low- and High arousal condition (e.g., [36,62,63]). Thereby, yellow steppe lemmings [63] showed no effect of arousal condition on USV call rate, whereas for fat-tailed gerbils [36] a higher call rate in the Isolation versus the Handling condition was documented. The increase in fundamental frequency in the High versus Low arousal condition is consistent with findings in yellow steppe lemmings [63] and fat-tailed gerbils [36]. Nevertheless, we must admit that, although we found significant differences in the acoustic parameters between both arousal conditions, these differences were not large enough to classify the calls significantly correctly to the Low or High arousal condition. Further, the differences between medians were small and the interquartile range showed a large overlap (Figure 3). Thus, playback studies will be needed to investigate whether these minor differences can elicit different responses from their caregivers.

**Figure 7:**
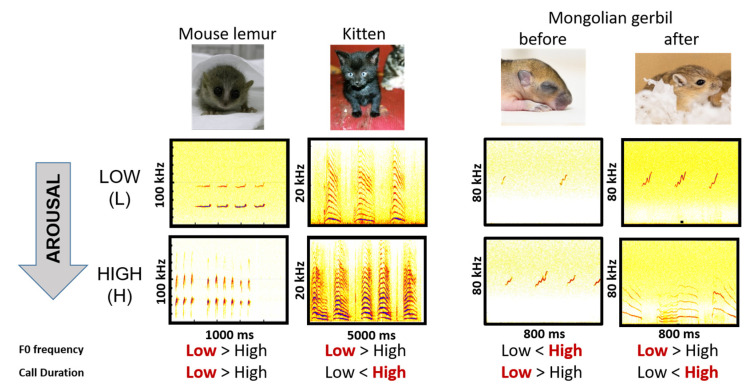
Comparison of vocalizations recorded in the Low versus High arousal conditions before and after eye opening for Mongolian gerbils with findings in the literature for mouse lemurs [26] and kittens [25].

Comparing the results of this study with our previous findings in mouse lemurs and kittens, using the same experimental protocol, revealed that before eye opening Mongolian gerbils produced the same call type (USV) in both arousal conditions, similar to our findings in kittens ([25], Figure 7). However, after eye opening an increased call rate of ADVs was only observed in the High arousal condition similar to our findings in gray mouse lemurs ([26]; Figure 7). Thereby, independent of whether the arousal conditions elicit the same or different call types, the direction of the acoustic differences was variable within and between species (Figure 7).

The hypothesis that, with increasing body weight and age, the call duration will increase while the fundamental and dominant frequency will decrease was not fulfilled in general. Moreover, it showed opposite patterns between call types. For USVs, call duration and fundamental frequency decreased with body weight and age following the common rules in relation to fundamental frequency but not call duration. A decrease in call duration and an increase of frequency with age was also found in other rodent species [62]. For ADVs, call duration and fundamental frequency increased following the common rules in relation to call duration but not fundamental frequency. However, our study is not the first that showed divergent patterns to the common rules. No effects of age or body weight on the fundamental frequency and/or call duration were reported in other mammalian infants and small-bodied mammals (e.g., fundamental frequency: speckled ground squirrels: [64], kittens: [25], both: Richardson ground squirrels: [65], tree shrews: [60]). A decrease of call duration with age was also found in other mammalian infants (e.g., piglets: [66], rodents: yellow steppe lemmings (USV: [67]; ADV: [18]), marmots: [16], fat-tailed gerbils: [17], mice: [62]) and can be explained by an increased independency from their caregivers. Ey et al. [12] argued that the effect of age and body size is more prominent in large-bodied species and less prominent in small-bodied species due to the different scale of variation. However, mixed results are even reported between call types within the same small-bodied species (e.g., piebald shrews: [40]; fat-tailed gerbils: [17]) and an increase of fundamental frequency is also reported for large-bodied animals (e.g., seals: [68]; deers: [69]).

The hypothesis that sender identity is dominantly encoded by filter-related parameters was not fulfilled in general. All acoustic parameters were individually distinct for USVs and ADVs, similar to other mammalian taxa (e.g., mice: [70]; see appendix [14]). For USVs, a discriminant function analysis supported that based on the acoustic parameters USVs can be correctly classified above chance level. Thereby, not only filter-related but also source- and tonality-related parameters were important for classification. This supports that in narrow-band tonal calls of high to ultrasonic fundamental frequencies individual distinctiveness is encoded by variation in the fundamental frequency (e.g., [19,20]).

The hypothesis that acoustic parameters will not differ between sexes was not fulfilled. Although the pups were not reproductively mature yet, we found an interaction between sex and age for USVs and sex-specific differences in the acoustic parameters, which differed between call types. While males produced USVs with a higher bandwidth than females, females produced ADVs with a higher fundamental frequency than males. For USVs, Holman and Seale [33] reported three sex-specific call types uttered during social encounter experiments in adult Mongolian gerbils. Whereas “Long rectilinear” and “Steep curvilinear” call types were only recorded during interactions involving males and were associated with sexual interactions, they reported the “Warble” only during female-female interactions testing gerbils older than 17 days. On the first view, this is in contrast to our study, where USVs, corresponding to the “Warble” reported by Holman and Seale [33], were also recorded from male pups. However, we have to note that Holman and Seale [33] self-stated that “We have unpublished data, however, that this type of vocalization is also made by preweaning (10–16 days) males.”. This suggests that the “Warble” occurred in male pups but seems to disappear in adult males, whereas it stays in the adult female repertoire. This sex-specific call usage is triggered by androgens, which seem to affect neural processing in the hypothalamus (e.g., [34,71]) early during ontogeny resulting in sex-specific vocal behavior in adult rodents (e.g., [53,72,73,74,75]). The sex-specific differences for ADVs might be related to differences in body weight or dominance, as shown in other mammalian species (e.g., [76,77]). However, in our study the body weight of the pups did not differ between sexes and the sex-specific parameters were not affected by body weight. In behavioral experiments we observed that in mixed sex interactions adult males are more dominant than adult females [78]. Thus, it needs to be investigated to which extent dominance affects the acoustic structure of gerbil vocalizations. All in all, our results suggest that males reduce the call rate of “Warbles” while developing “Long rectilinear” and “Steep curvilinear” call types and potentially provide ADVs with lower fundamental frequency to signal their dominance or mate quality.

### 4.3. How Divergent Encoding Patterns Can Be Explained?

Our results showed that acoustic variations related to arousal and physical characteristics do not always follow the common rules proposed for mammals. For arousal, differences between species might be explained by different experiences of the arousal conditions. For example, differences in the response to social isolation from the caregiver were also reported for two closely related vole species (prairie versus montane vole; [79]). Prairie voles emitted a high rate of vocalizations during isolation, which were correlated with their plasma corticosterone level, whereas montane voles emitted no isolation calls and showed only a minor increase of cortisol level. Thus, even if we use the same experimental design, it can induce or increase different arousal levels in different species, indicating species-specific anxiety-related responses at an early developmental stage [80]. Furthermore, the experience of arousal might also be dependent from the developmental state, especially from the sensory input or the development of neuronal control mechanisms. Thus, opening the eyes might change the perception of discomfort (which could also be a result of ruff handling from the caregivers) to the perception of a threat stimulus (predator). Thus, a switch of call type might not only reflect an increase in arousal but also a different function of the vocalization.

Divergent encoding patterns were not only found between species but also between call types within the same species (e.g., [21]; see appendix [14]). Although the anatomy of the vocal tract system seems to be evolutionarily conserved in mammals, vocal production mechanisms can differ between, and even within, a species depending on the call type (e.g., [38,39]). Thus, it can be assumed that the structure of the call type and the related production system might be affected by the physiological and anatomical induced changes. For rodents, two different mechanisms in vocal production are suggested depending on the call type [81]. Audible calls (ADV) are suggested to be produced by vocal fold vibrations, whereas ultrasonic calls (USV) are suggested to be produced by an additional anatomical structure, a ventral pouch in the larynx-area (=whistle mechanism; [38]). These variations in the vocal production between call types are suggested to explain different coding patterns for different call types. Furthermore, the opposite patterns of USVs and ADVs encoding body size and age may be explained by different growing patterns of the different structures of the vocal apparatus [40].

## 5. Conclusions

Mongolian gerbil pup vocalizations encode the arousal and developmental state of the sender as well as identity and sex. Comparing USV and ADV rates, we found that USVs encode a strongly dependent pup, while ADVs signal a more high-aroused matured pup. Thereby, the coding pattern differed between USVs and ADVs, which can be explained by different production mechanisms (whistle mechanism/vocal folds). Both call types do not always fulfill the proposed common rules for mammals to encode arousal and physical characteristics. We conclude that vocal communication is too complex to just follow simple rules. The rules might be adapted to several factors which can influence the vocal behavior of mammalian infants towards the same contexts even in closely related species. Thus, further comparative studies are needed to clarify to which extent socio-ecological factors (e.g., predator, breeding system) as well as anatomical and neuronal constrains of the vocal production systems (e.g., vocal production mechanism, neuronal perception and motor control) shape the encoding of arousal and physical characteristics of the sender in mammals.

## Figures and Tables

**Table 1:** Description of measured acoustic parameters. Time frame for the analysis of source-related parameters = 3 ms; Abb. = Abbreviations.

Acoustic Parameter	Abb.	Definition
Time-related parameters		
Call duration [ms]	Dur	Time between the onset and the offset of a call.
Time of minimum fundamental frequency [ms]	TimeminF0	Time between the onset and the time point of minimum fundamental frequency of a call.
Time of maximum fundamental frequency [ms]	TimemaxF0	Time between the onset and the time point of maximum fundamental frequency of a call.
Source-related parameters		
Minimum fundamental frequency [kHz]	MinF0	Lowest value of the fundamental frequency across all time frames of a call.
Maximum fundamental frequency [kHz]	MaxF0	Highest value of the fundamental frequency across all time frames of a call.
Bandwidth [kHz]	BandF0	MaxF0–MinF0.
Mean fundamental frequency [kHz]	MeanF0	Mean fundamental frequency of a call calculated across all time frames of a call.
Standard deviation of fundamental frequency [kHz]	SDF0	Standard deviation of the fundamental frequency of a call calculated across all time frames of a call.
Meanslope [kHz/s]	SlopeF0	Mean absolute slope of the fundamental frequency calculated as the sum of the absolute difference of the F0 of two consecutive time frames.
Filter-related parameters		
Center of gravity [kHz]	CoG	Mean frequency of the spectrum of a call weighted by the amplitude of a call.
Standard deviation of CoG [kHz]	SD	Standard deviation of the CoG measuring the deviation of frequency values from the CoG of a call.
Skewness	Ske	Difference between the spectral distribution below and above the CoG of a call.
Kurtosis	Kur	Difference between the spectral distribution around the CoG from a Gaussian distribution of a call.
Tonality-related parameters		
Voiced percentage [%]	Voiced	Percentage of voiced time frames of a call.
Harmonics-to-noise-ratio [dB]	Hnr	Ratio between the periodic (harmonic part) and aperiodic (noise) components of a call.
Wiener entropy [dB]	Entropy	Ratio of geometric to arithmetic energy of a call.

**Table 2:** Mean and standard deviation of the acoustic parameters for clusters I to III; the corresponding call type is indicated in brackets.

Acoustic Parameter	Cluster I (USV-ADV)	Cluster II (USV)	Cluster III (ADV)
Time-related parameters			
Dur [ms]	109.0 ± 31.1	89.1 ± 32.3	93.6 ± 65.1
TimeminF0 [ms]	101.0 ± 31.0	9.6 ± 12.0	77.5 ± 63.7
TimemaxF0 [ms]	72.8 ± 27.7	81.6 ± 32.2	14.6 ± 25.5
Source-related parameters			
MinF0 [kHz]	4.6 ± 4.0	36.5 ± 3.3	6.1 ± 4.7
MaxF0 [kHz]	51.9 ± 4.3	49.3 ± 5.0	11.3 ± 5.8
BandF0 [kHz]	47.3 ± 5.7	12.9 ± 4.4	5.2 ± 4.0
MeanF0 [kHz]	38.3 ± 6.3	42.7 ± 3.4	8.5 ± 4.8
SDF0 [kHz]	15.6 ± 3.9	3.4 ± 1.3	1.6 ± 1.2
SlopeF0 [kHz/s]	758.7 ± 282.3	382.8 ± 177.5	98.5 ± 57.3
Filter-related parameters			
CoG [kHz]	34.7 ± 10.5	44.0 ± 3.6	10.8 ± 6.5
SD [kHz]	15.7 ± 5.7	5.5 ± 2.4	9.1 ± 2.6
Ske	−0.9 ± 1.8	4.5 ± 2.7	2.8 ± 1.7
Kur	5.4 ± 11.6	46.7 ± 53.4	10.8 ± 13.6
Tonality-related parameters			
Voiced [%]	89.2 ± 13.8	94.5 ± 6.8	85.6 ± 19.2
Hnr [dB]	8.2 ± 5.9	12.5 ± 3.0	8.7 ± 4.5
Entropy [dB]	−4.7 ± 1.3	−0.8 ± 0.9	−6.2 ± 1.4

**Table 3:** Results of the final GLMM and LME models testing the effects of Age group, Arousal, Sex, and Order on the call occurrence and call rates of three different call types; bold *p*-values represent significant difference *p* < 0.05, df = degree of freedoms.

	Call Occurrence			Call Rate		
Predictors	χ^2^	df	*p*-Value	χ^2^	df	*p*-Value
USV						
Age group	35.36	3	**<0.001**	100.57	3	**<0.001**
Arousal	3.59	1	0.058	0.71	1	0.399
Sex	0.07	1	0.788	0.22	1	0.641
Order	0.02	1	0.883	0.02	1	0.887
Age group * Sex				10.68	3	**0.014**
ADV						
Age group	6.48	3	0.090	17.43	3	**<0.001**
Arousal	10.33	1	**0.001**	6.24	1	**0.013**
Sex	0.55	1	0.458	1.51	1	0.220
Order	0.33	1	0.566	0.31	1	0.577
Age group * Arousal				14.48	3	**0.002**
USV-ADV						
Age group	10.82	3	**0.013**	11.33	3	**0.010**
Arousal	5.43	1	**0.020**	0.003	1	0.960
Sex	1.26	1	0.263	1.22	1	0.269
Order	0.06	1	0.809	1.32	1	0.250
Age group * Sex				7.92	3	**0.048**

**Table 4:** Summary of the results of the LME analysis testing acoustic parameters for effects of Arousal, Body weight, Age group, Sender identity and Sex. USV = ultrasonic vocalizations; ADV = audible vocalizations; **L** > H: Values for Low arousal larger than for High arousal; L < **H**: Values for High arousal larger than for Low arousal; **↓**: values decreased with age; **↑**: values increased with age; + = significant effect; **F** > M: Values larger for females than for males; F < **M**: Values larger for males than for females; blank cells = no significant difference.

Parameters	Arousal	Body Weight		Age Group		Identity		Sex	
	USV	USV	ADV	USV	ADV	USV	ADV	USV	ADV
Time-related parameters									
Duration	L > H	**↓**	**↑**	**↓**	**↑**	+	+		
TimeminF0	L > H		**↑**		**↑**	+	+		
TimemaxF0		**↓**		**↓**		+	+		F > M
Source-related parameters									
MinF0	L < H	**↓**		**↓**		+	+		F > M
MaxF0	L < H	**↓**		**↓** **↑**	**↑**	+	+		
BandF0				**↓** **↑**	**↑**	+	+	F < M	
MeanF0	L < H	**↓**		**↓**		+	+		F > M
SDF0	L < H	**↑**		**↓** **↑**	**↑**	+	+		
SlopeF0		**↑**		**↑**		+	+		
Filter-related parameters									
CoG	L < H	**↓**		**↓**		+	+		F > M
SD	L > H	**↑**				+	+		
Ske	L > H	**↓**		**↓**	**↓**	+	+		
Kur						+	+		
Tonality-related parameters									
Voiced			**↓**	**↑**	**↓**	+	+		
Hnr	L < H			**↑**		+	+		
Entropy	L > H					+	+		

## Data Availability

Raw data will be available from the data repository Mendeley Data: Silberstein, Yara; Felmy, Felix; Scheumann, Marina (2023), “Dataset: Encoding of Arousal and Physical Characteristics in Audible and Ultrasonic Vocalizations of Mongolian Gerbil Pups Testing Common Rules for Mammals”, Mendeley Data, V1, doi:10.17632/y6yx925663.1. Audio and video data are stored at the Institute of Zoology and are available on reasonable request.

## Supplementary Materials

The following supporting information can be downloaded at: https://www.mdpi.com/article/10.3390/ani13162553/s1, Figure S1. Correlation plots and Pearson correlation coefficients (R) between the acoustic parameters of all calls. Figure S2. Plot showing the optimum number of clusters based on the Elbow figure produced by the Silhouette method (general unsupervised clustering; K-means model). Figure S3. Boxplots for (A) USV rate, (B) ADV and (C) USV-ADV rate for pups which produced the respective call type in each Age group. Table S1. Results of the LME models testing the effect of Arousal on USV acoustic parameters. Table S2. Results of the LME models testing the effect of Body weight on USV and ADV acoustic parameters. Table S3. Results of the LME models testing effects for Age group on acoustic parameters of USVs and ADVs. Table S4. Results of the LME models testing the effect of Individual on USV and ADV acoustic parameters. Table S5. Results of the LME models testing the effect of Sex on USV and ADV acoustic parameters.
