# Supplementary material for: Encoding of Arousal and Physical Characteristics in Audible and Ultrasonic Vocalizations of Mongolian Gerbil Pups Testing Common Rules for Mammals
Source: Animals (Basel). 2023 Aug 8;13(16):2553. doi: 10.3390/ani13162553 (PMC10451175; doi:10.3390/ani13162553)
Supplement: Supplementary file 1 [file animals-13-02553-s001.zip › animals-2521336-supplementary.pdf]

Supplementary materials for

Encoding of Arousal and Physical Characteristics in Audible and Ultrasonic Vocalizations of Mongolian Gerbil Pups Testing Common Rules for Mammals

Yara Silberstein et al.

* Corresponding author: yara.silberstein@tiho-hannover.de

This PDF file contains:

Figures S1 to S3
Tables S1 to S5

Figure S1.
Correlation plots and Pearson correlation coefficients (R) between the acoustic parameters of all calls.

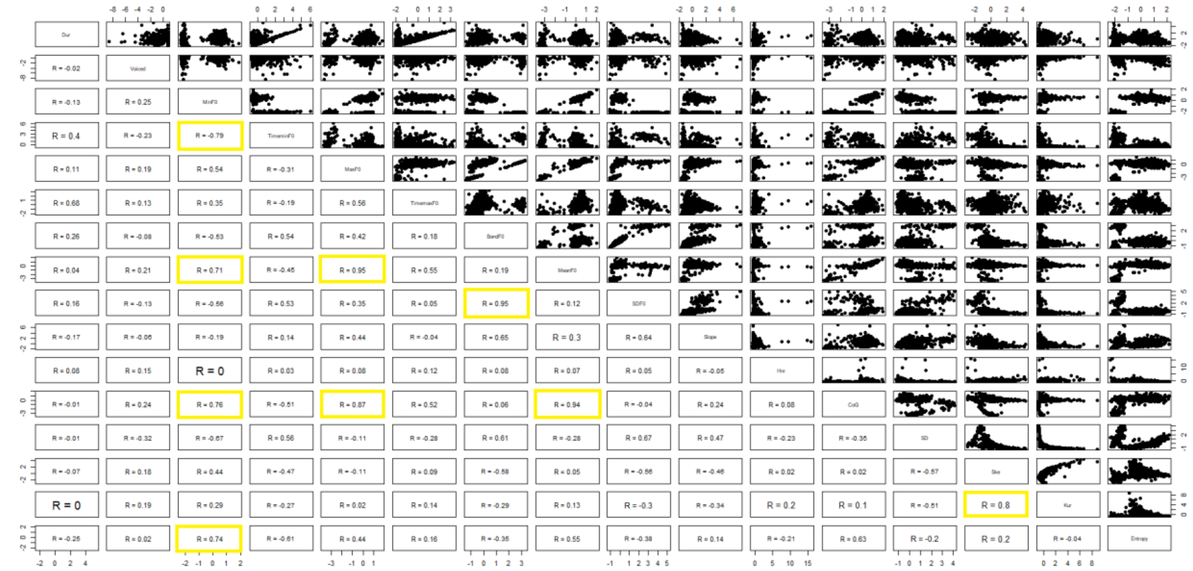

Figure S2.
Plot showing the optimum number of clusters based on the Elbow figure produced by the Silhouette method (general unsupervised clustering; K-means model).

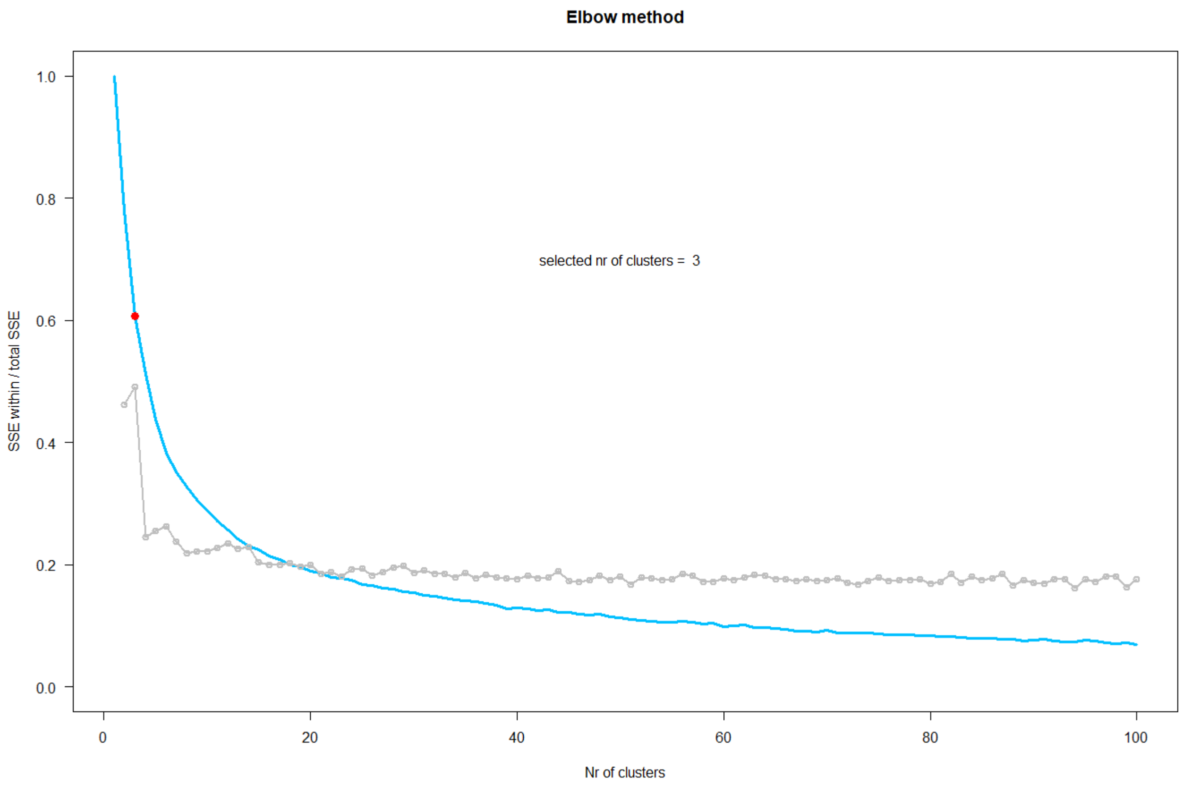

Figure S3.

Boxplots for (A) USV rate, (B) ADV and (C) USV-ADV rate for pups which produced the respective call type in each Age group. Boxplots represent lower and upper quartile, thick black line is the median, whiskers are non-outlier range, yellow dots represent number of vocalizations plotted as a beeswarm.

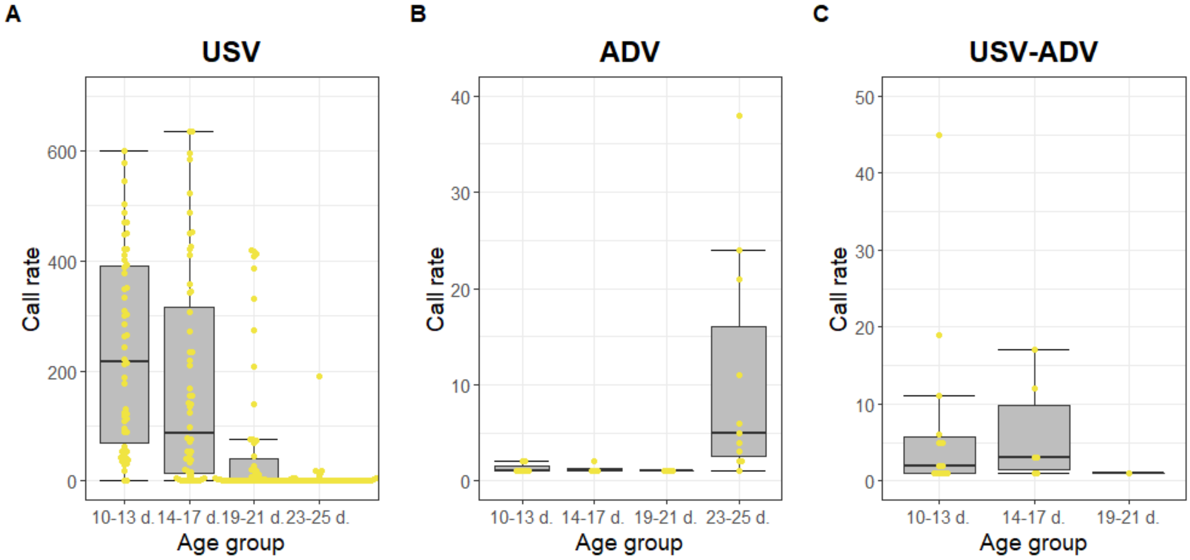

Table S1.

Results of the LME models testing the effect of Arousal on USV acoustic parameters; df = 653, SE: standard error; Bold: $p < 0.05$

Parameter	Estimate	SE	t-value	p-value
Time-related parameters				
Duration	8.14	2.28	3.57	**<0.001**
TimeminF0	2.26	0.92	2.46	**0.014**
TimemaxF0	3.88	2.24	1.73	0.084
Source-related parameters				
MinF0	-1.13	0.22	-5.07	**<0.001**
MaxF0	-1.61	0.33	-4.91	**<0.001**
BandF0	-0.44	0.32	-1.40	0.161
MeanF0	-1.08	0.21	-5.13	**<0.001**
SDF0	-0.25	0.09	-2.80	**0.005**
SlopeF0	14.04	12.17	1.15	0.249
Filter-related parameters				
CoG	-1.05	0.21	-4.97	**<0.001**
SD	0.39	0.15	2.57	**0.011**
Ske	0.53	0.19	2.85	**0.005**
Kur	-5.66	3.74	-1.51	0.131
Tonality-related parameters				
Voiced	0.50	0.51	0.97	0.332
Hnr	-2.69	1.12	-2.42	**0.016**
Entropy	0.03	0.01	2.87	**0.004**

Table S2

Results of the LME models testing the effect of Body weight on USV and ADV acoustic parameters. SE: standard error; Bold: $p < 0.05$

	USV (df = 653)				ADV (df = 47)			
Parameter	Estimate	SE	t-value	p-value	Estimate	SE	t-value	p-value
Time-related parameters								
Duration	-4.25	0.52	-8.12	**<0.001**	4.80	1.99	2.41	**0.020**
TimeminF0	0.01	0.21	0.03	0.976	4.31	1.91	2.26	**0.029**
TimemaxF0	-4.40	0.51	-8.60	**<0.001**	0.57	0.68	0.84	0.408
Source-related parameters								
MinF0	-0.59	0.05	-12.53	**<0.001**	-0.04	0.07	-0.57	0.574
MaxF0	-0.44	0.08	-5.60	**<0.001**	0.14	0.11	1.29	0.204
BandF0	0.09	0.07	1.26	0.209	0.19	0.11	1.77	0.083
MeanF0	-0.55	0.05	-11.33	**<0.001**	0.03	0.08	0.39	0.697
SDF0	0.05	0.02	2.31	**0.021**	0.06	0.03	1.68	0.099
SlopeF0	38.99	2.53	15.39	**<0.001**	-2.49	1.89	-1.32	0.195
Filter-related parameters								
CoG	-0.35	0.05	-6.77	**<0.001**	-0.01	0.13	-0.09	0.928
SD	0.15	0.04	4.23	**<0.001**	-0.04	0.06	-0.60	0.550
Ske	-0.09	0.04	-2.11	**0.035**	-0.04	0.05	-0.78	0.437
Kur	-0.90	0.89	-1.01	0.311	-0.15	0.65	-0.24	0.812
Tonality-related parameters								
Voiced	0.15	0.11	1.32	0.187	-1.59	0.53	-2.98	**0.005**
Hnr	0.13	0.27	0.47	0.638	-0.24	0.76	-0.32	0.750
Entropy	0.00	0.00	1.09	0.277	0.00	0.00	1.33	0.191

Table S3.

Results of the LME models testing effects for Age group on acoustic parameters of USVs and ADVs. For USVs results of the Anova of the LME model are represented comparing Age group 10-13 d., 14-17 d. and 19-21 d. For ADVs results of the LME model are represented comparing the combined Age groups 10-21 d. with Age group 23-25 d. If the predictor had more than two levels, we reported the results of the ANOVA of the model. If the predictor had only two levels, we reported the estimates and standard error together with the results of the t-test. SE: standard error; Bold: $p < 0.05$

	USV (df = 2)		ADV (df = 47)			
Parameter	χ^2	p-value	Estimate	SE	t-value	p-value
Time-related parameters						
Duration	130.04	**<0.001**	67.16	23.07	2.91	**0.006**
TimeminF0	2.17	0.338	60.42	22.45	2.69	**0.010**
TimemaxF0	161.27	**<0.001**	11.38	9.49	1.25	0.219
Source-related parameters						
MinF0	50.54	**<0.001**	0.03	0.73	0.04	0.967
MaxF0	60.52	**<0.001**	3.88	1.34	2.88	**0.006**
BandF0	44.32	**<0.001**	3.76	1.35	2.79	**0.008**
MeanF0	122.06	**<0.001**	1.63	0.94	1.74	0.088
SDF0	40.42	**<0.001**	0.92	0.44	2.06	**0.045**
SlopeF0	219.48	**<0.001**	-23.85	21.34	-1.12	0.269
Filter-related parameters						
CoG	70.30	**<0.001**	1.52	1.57	0.97	0.337
SD	0.99	0.608	0.81	0.92	0.88	0.384
Ske	7.59	**0.022**	-1.32	0.53	-2.48	**0.017**
Kur	0.82	0.663	-1.20	5.67	-0.21	0.833
Tonality-related parameters						
Voiced	19.58	**<0.001**	-16.51	7.17	-2.30	**0.026**
Hnr	12.61	**0.002**	-1.45	5.53	-0.26	0.794
Entropy	2.70	0.259	0.01	0.01	0.85	0.398

Table S4.
Results of the LME models testing the effect of Individual on USV and ADV acoustic parameters. SE: standard error; Bold: $p < 0.05$

	USV (df = 25)		ADV (df = 12)	
Parameter	χ^2	p-value	χ^2	p-value
Time-related parameters				
Duration	235.32	**<0.001**	66.67	**<0.001**
TimeminF0	92.11	**<0.001**	61.62	**<0.001**
TimemaxF0	276.12	**<0.001**	21.26	**0.047**
Source-related parameters				
MinF0	384.13	**<0.001**	80.42	**<0.001**
MaxF0	463.74	**<0.001**	26.78	**0.008**
BandF0	245.66	**<0.001**	31.27	**0.002**
MeanF0	668.63	**<0.001**	39.74	**<0.001**
SDF0	255.46	**<0.001**	34.93	**<0.001**
SlopeF0	304.50	**<0.001**	84.94	**<0.001**
Filter-related parameters				
CoG	726.77	**<0.001**	39.71	**<0.001**
SD	492.55	**<0.001**	22.62	**0.031**
Ske	290.34	**<0.001**	47.65	**<0.001**
Kur	239.05	**<0.001**	111.72	**<0.001**
Tonality-related parameters				
Voiced	114.42	**<0.001**	37.65	**<0.001**
Hnr	195.61	**<0.001**	258.49	**<0.001**
Entropy	366.30	**<0.001**	36.83	**<0.001**

Table S5.

Results of the LME models testing the effect of Sex on USV and ADV acoustic parameters. SE: standard error; Bold: $p < 0.05$

	USV (df = 18)				ADV (df = 6)			
Parameter	Estimate	SE	t-value	p-value	Estimate	SE	t-value	p-value
Time-related parameters								
Duration	8.75	5.21	1.68	0.110	-18.11	29.68	-0.61	0.564
TimeminF0	-0.29	1.67	-0.17	0.864	-14.78	28.00	-0.53	0.616
TimemaxF0	9.37	5.19	1.81	0.088	-17.10	6.94	-2.46	**0.049**
Source-related parameters								
MinF0	-0.42	0.54	-0.77	0.452	-2.25	0.57	-3.99	**0.007**
MaxF0	1.30	1.03	1.26	0.223	-1.73	1.45	-1.19	0.278
BandF0	1.82	0.78	2.32	**0.032**	0.04	1.56	0.26	0.805
MeanF0	0.45	0.82	0.55	0.591	-2.46	0.78	-3.15	**0.020**
SDF0	0.48	0.23	2.10	0.050	0.31	0.48	0.63	0.550
SlopeF0	54.02	28.59	1.89	0.075	25.87	25.29	1.02	0.346
Filter-related parameters								
CoG	0.51	0.89	0.58	0.571	-4.74	0.96	-4.93	**0.003**
SD	-0.13	0.44	-0.29	0.773	-1.38	0.71	-1.96	0.098
Ske	-0.82	0.54	-1.51	0.148	0.89	0.42	2.10	0.080
Kur	-12.54	9.43	-1.33	0.200	-4.40	9.61	-0.46	0.663
Tonality-related parameters								
Voiced	1.00	0.95	1.05	0.308	-3.00	6.12	-0.49	0.641
Hnr	2.39	2.46	0.97	0.344	-20.82	12.62	-1.65	0.150
Entropy	-0.03	0.03	-0.79	0.442	0.01	0.01	0.42	0.691
